# Machine-learning models for Alzheimer’s disease diagnosis using neuroimaging data: survey, reproducibility, and generalizability evaluation

**DOI:** 10.1186/s40708-025-00252-3

**Published:** 2025-03-21

**Authors:** Maryam Akhavan Aghdam, Serdar Bozdag, Fahad Saeed, Alzheimer’s Disease Neuroimaging Initiative

**Affiliations:** 1 Knight Foundation School of Computing and Information Science (KFSCIS), Florida International University (FIU), Miami, FL USA; 2 Department of Computer Science and Engineering, University of North Texas (UNT), Denton, TX USA

**Keywords:** Alzheimer's disease, Machine learning, Deep learning, Structural MRI, Functional MRI, PET, Reproducibility, Generalizability

## Abstract

Clinical diagnosis of Alzheimer’s disease (AD) is usually made after symptoms such as short-term memory loss are exhibited, which minimizes the intervention and treatment options. The existing screening techniques cannot distinguish between stable MCI (sMCI) cases (i.e., patients who do not convert to AD for at least three years) and progressive MCI (pMCI) cases (i.e., patients who convert to AD in three years or sooner). Delayed diagnosis of AD also disproportionately affects underrepresented and socioeconomically disadvantaged populations. The significant positive impact of an early diagnosis solution for AD across diverse ethno-racial and demographic groups is well-known and recognized. While advancements in high-throughput technologies have enabled the generation of vast amounts of multimodal clinical, and neuroimaging datasets related to AD, most methods utilizing these data sets for diagnostic purposes have not found their way in clinical settings. To better understand the landscape, we surveyed the major preprocessing, data management, traditional machine-learning (ML), and deep learning (DL) techniques used for diagnosing AD using neuroimaging data such as structural magnetic resonance imaging (sMRI), functional magnetic resonance imaging (fMRI), and positron emission tomography (PET). Once we had a good understanding of the methods available, we conducted a study to assess the reproducibility and generalizability of open-source ML models. Our evaluation shows that existing models show reduced generalizability when different cohorts of the data modality are used while controlling other computational factors. The paper concludes with a discussion of major challenges that plague ML models for AD diagnosis and biomarker discovery.

## Introduction

Alzheimer's disease and related dementias (ADRD) is a growing public health crisis, affecting about 50 million people around the world, and 6 million people in the US (expected to rise to 14 million by 2050), leading to huge burden to patients, caretakers, and the healthcare sector ($250 Billion in the US alone) [1, 2]. The current clinical screening for AD/ADRD is mainly based on cognitive, neurological, functional, and behavioral tests. A combination of tests, including amyloid and tau biomarkers, cerebrospinal fluid, and cognitive tests, such as the Mini-Mental State (MMSE) Examination and Clinical Dementia Rating (CDR), [3] are some of the methods currently clinically used. These tests are shown to exhibit variable performance for ADRD diagnosis depending on the kind of dementia, race, age, and other factors. Perhaps the major limitation of these methods is that they are executed after symptoms have appeared, making early intervention almost impossible. Early interventions may lead to better outcomes for the patients and their families, and therefore, identifying people at an early stage of AD is crucial and of significant impact—especially for under-represented and socioeconomically disadvantaged populations [1].

ADRD is a heterogeneous and complex disorder, with a wide spectrum of variations between healthy brains and AD. Despite differences in the precise definition, most scientists believe that mild cognitive impairment (MCI) is an intermediate stage between cognitively unimpaired (CU) and dementia cases where people with MCI could experience memory and language problems but can manage their day-to-day activities. Further, certain people diagnosed with MCI do not progress to AD immediately, and others experience a rapid progression [4]. These two cases are called stable MCI (sMCI) and progressive MCI (pMCI), respectively. By some estimates, approximately 12–18% of people aged 60 or older suffer from MCI, and 10–15% of people with MCI are more likely to develop AD each year [5]. Discovering biomarkers specific to sMCI and pMCI could lead to early AD detection. However, the identification of effective biomarkers remains a considerable challenge, which is complicated by heterogeneity in MCI/AD, patient demographics, and highly variable and non-linear symptom patterns of MCI/AD [6].

High-throughput neuroimaging data could potentially be used for early identification and diagnosis of AD. However, the high dimensionality of the data poses a significant challenge and requires advanced computational methods to make inferences and predictions. Machine learning (ML) is a branch of artificial intelligence (AI) that has been proven to process high-dimensional neuroimaging data and detect complex patterns beyond the capabilities of human analysis. Various ML approaches have been successful in diagnosing brain disorders, including Autism Spectrum Disorder (ASD) [7–10], Parkinson’s disease [11–13]. ML-based characterization, diagnosis, and prediction of AD using neuroimaging data [14–18] is an active area of research and, therefore there is an increased interest in survey papers, and empirical studies that can capture the current state of the art. Such studies are useful for current practitioners, and more importantly to new entrants in the field, including students and scientists. Indeed, there are two surveys that were recently published [19, 20]. Khojaste-Sarakhsi et al.'s work [20] is focused on deep-learning methods, including convolutional neural network (CNN), autoencoder (AE), recurrent neural network (RNN), restricted Boltzmann machine (RBM), and deep belief network (DBN). However, they do not include traditional ML methods in their study which might be useful for comparisons with DL methods. Their study also does not include data analysis strategies and pre-processing pipelines, which may significantly affect the performance of ML models. Gao and Lima [19] attempted to fill this gap by incorporating preprocessing pipelines in their comprehensive review. However, traditional ML methods (which are large in number) were not integrated and they may not provide vital information to new practitioners in the field. This paper is also more comprehensive since we investigate the reproductivity and generalizability of existing open-source ML models.

In this study, we attempt to fill the gaps of existing surveys (Table 1) and identify research challenges that can be tackled by students, and new entrants in the field. Our study makes the following contributions:

1. *Comprehensive modality coverage*: Unlike prior surveys, this study incorporates all major neuroimaging modalities, including structural MRI (sMRI), functional MRI (fMRI), and positron emission tomography (PET), as well as combinations of these modalities (e.g., sMRI & PET, sMRI & fMRI). This provides a holistic view of data sources and their role in AD diagnosis.
2. *Emphasis on preprocessing and data analysis strategies*: While previous works often overlook the influence of preprocessing pipelines, this study delves into voxel-based, slice-based, ROI-based, and patch-based methods. The impact of widely used preprocessing tools such as FSL, FreeSurfer, and SPM is evaluated, ensuring that practitioners understand how these steps affect ML model performance.
3. *Integration of traditional and deep learning methods*: Existing surveys, such as Khojaste-Sarakhsi et al.[20], focus exclusively on deep learning methods. This study bridges the gap by providing a comprehensive review of both traditional ML and deep learning techniques, facilitating comparisons, and offering valuable insights to both seasoned researchers and newcomers.
4. *Reproducibility and generalizability evaluations*: A key distinguishing feature of this work is the empirical evaluation of open-source ML models for reproducibility and generalizability. By replicating experiments with the same preprocessing pipelines and data cohorts, and by assessing model performance across different datasets, this study addresses a critical barrier to clinical adoption.

**Table 1:** Comparison between existing literature surveys and this study

Study		[20]	[19]	This study
Modality	sMRI	Y	Y	Y
	fMRI	Y	Y	Y
	PET	Y	Y	Y
Pre-processing pipelines		–	Y	Y
Data analysis strategies (VB: Voxel-based; SB: Slice-based; RB: ROI-based; PB: Patch-based)	VB	ND	Y	Y
	SB	ND	Y	Y
	RB	ND	Y	Y
	PB	ND	Y	Y
Traditional ML	RF	–	–	Y
	SVM	–	–	Y
Deep-Learning Methods	CNN	Y	Y	Y
	AE	Y	Y	Y
	DBN	Y	Y	Y
	RBM	Y	Y	Y
	RNN	Y	Y	Y
	ViT	–	–	Y
Model Reproducibility evaluation		–	–	Y
Model Generalizability evaluation		–	–	Y

*Y* Yes, *ND* Not described in detail, *RF* Random Forest, *SVM* Support Vector Machine, *CNN* Convolutional Neural Network, *AE* Auto-Encoder, *DBN* Deep Belief Network, *RBM* Restricted Boltzmann Machine, *RNN* Recurrent Neural Network, *ViT* Vision Transformer

We believe that this paper is substantially different from surveys [19, 20] (as illustrated in Table 1), and adds immense value that can be used by ML and clinical scientists alike.

The rest of this review is organized as follows: In Sects. 2.1, 2.2, and 2.3, we explain neuroimaging modalities, data sources, and necessary pre-processing steps, respectively. In Sect. 2.4, we discuss data analysis strategies. Section 2.5 describes the traditional ML and DL models used for AD diagnosis. In Sect. 3, we perform a comparative analysis of the deep-learning models for AD. In Sect. 4, we report on a reproducibility and generalizability study of existing open-source models. Section 5 concludes the paper with a discussion on the current state of the art in the field, and in Sect. 6, we provide future directions.

## Material and methods

There are various key components that work together to form a diagnostic method for ADRD. Each of the components informs or affects the overall accuracy, reliability, and efficiency of the ML model. The following section describes each of those components with a commentary on the state of art in that field of work.

### Neuroimaging modalities

This section provides a brief overview of the neuroimaging modalities that could be used for the characterization of ADRD. While the number of neuroimaging modalities is large, we will focus on structural MRI (sMRI), functional MRI (fMRI) and positron emission tomography (PET) that have been frequently used by ML models.

*The structural MRI (sMRI)* MRI is a non-invasive imaging technique that provides exquisite detail of brain, spinal cord, and vascular anatomy and can visualize anatomy in all three planes: axial, sagittal, and coronal of the brain's structure. Magnetic resonance imaging (MRI) leverages the inherent magnetic properties of atomic nuclei. A potent, homogenous external magnetic field is applied to induce alignment of the typically randomly oriented protons within the water molecules of the target tissue. The established nuclear alignment, or magnetization, is subsequently perturbed by the introduction of an external radiofrequency (RF) pulse. By strategically manipulating the sequence and timing of these applied and received RF pulses, distinct image contrasts can be generated. Repetition Time (TR) signifies the interval between consecutive pulse sequences targeting the same tissue slice. Conversely, Time to Echo (TE) defines the duration between the application of the RF pulse and the acquisition of the corresponding echo signal. Most common sMRI sequences include T1-weighted (longitudinal relaxation time) scans which are produced using short TE and TR times; T2-weighted (transverse relaxation time) scans which are produced using longer TE and TR times. Differentiation between T1 and T2 weighted scans can be done using CSF contrast which is dark for T1 weighted and bright for T2 weighted [21].

*The functional MRI (fMRI)* Functional magnetic resonance imaging (fMRI) utilizes the blood-oxygen-level-dependent (BOLD) contrast to indirectly map neural activity. Using established MRI principles, a strong magnetic field aligns tissue protons, and radio wave pulses at specific frequencies induce energy absorption and realignment of these protons. When radio wave cessation occurs, the protons release the absorbed energy which generates a readable signal that can be used. The blood-oxygen-level-dependent (BOLD) contrast exploits the differing magnetic properties of oxygenated hemoglobin (oxyHb) compared to deoxygenated hemoglobin (deoxyHb). The implicit assumption that increased neural activity is correlated with increased oxygenated blood flow will lead to localized shifts in the magnetic field due to increased oxyHb. These BOLD signal changes are captured using pulse sequences, which are reconstructed in a 3-dimensional image. By detecting changes in blood flow, fMRI helps researchers understand which brain areas are active during specific tasks or cognitive processes [21].

*The PET* Positron emission tomography (PET) is a nuclear medicine imaging technique that uses a small amount of a radioactive tracer (radiopharmaceutical) to visualize molecular processes in the body. In brain imaging, PET scans can measure metabolic activity, blood flow, and specific neurotransmitter receptor bindings. It is commonly used to study brain metabolism and to assess changes associated with neurodegenerative disorders like AD [21].

**Fig. 1:**
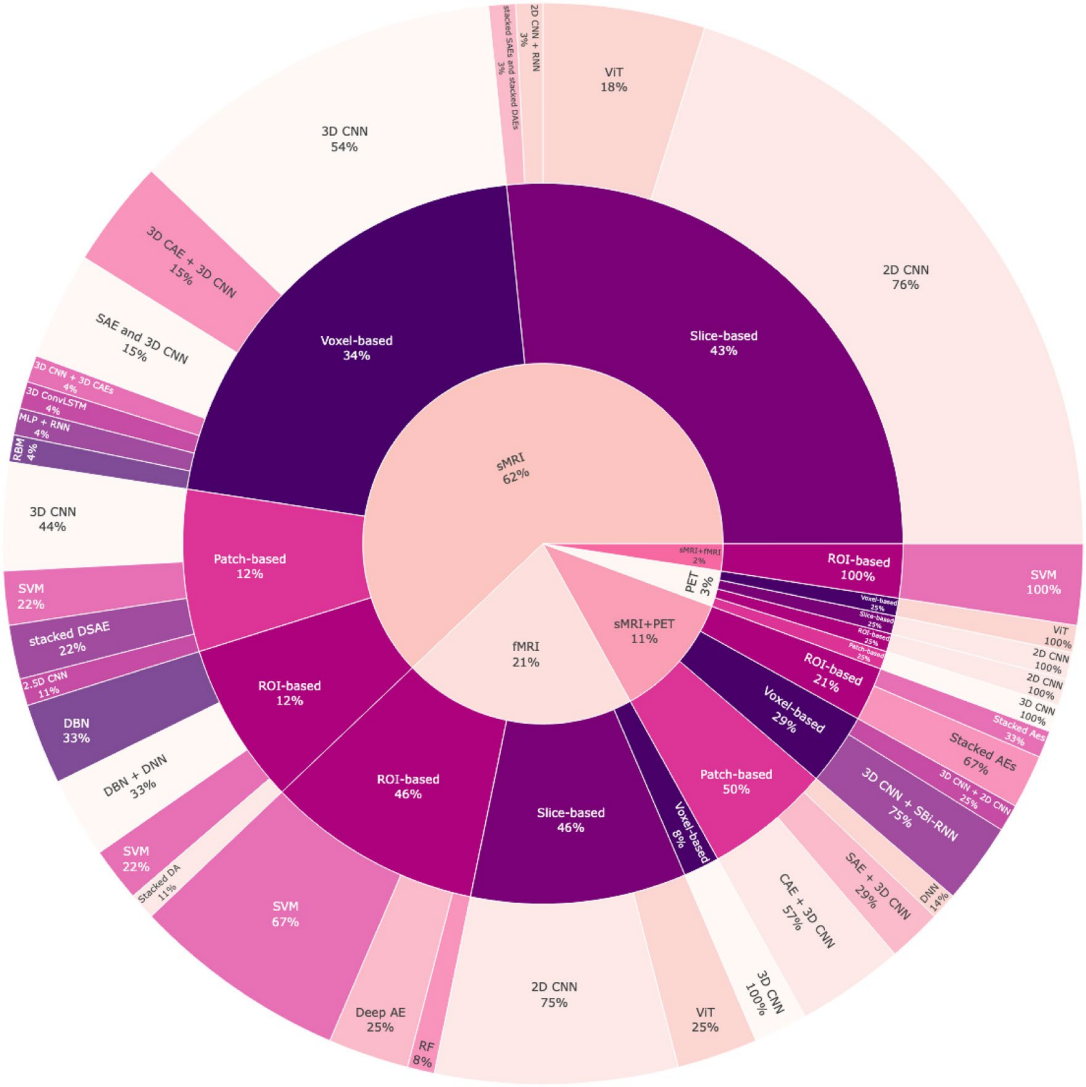
Overview of our survey paper based on modalities, data analysis strategies, classification methods, and tasks on the ADNI dataset. This figure provides a comprehensive birds-eye view of the distribution of papers across different modalities (e.g., sMRI, fMRI), data analysis systems (e.g., voxel-based, ROI-based), and machine learning models (e.g., CNN, ViT). It highlights the fraction of studies focusing on each combination, offering insight into current trends and research focus areas in ADNI-based studies

Since each technique provides unique information about brain structure and function, researchers have used all these modalities to develop computational techniques to identify signatures that can be used for the classification of MCI and AD. While each technique provides unique information about the brain's structure or function and is crucial in investigating neurological conditions and studying brain activity, it was unclear what fraction of modality was being used for developing machine-learning models. Figure 1 provides a clear birds-eye-view picture of what fraction of papers are focusing on different modalities, different data analysis systems, various kinds of ML models, and what classification classes are being used. Each of the different levels will be referenced in the appropriate sections of the paper. Figure 1 (first level) represents the percentage of reviewed papers using these neuroimaging modalities across 70 papers and shows the research community's interest in specific modalities due to their perceived efficacy, availability, and relevance in AD research. As can be seen in the figure, sMRI stands out as the most utilized modality, comprising 63% of the surveyed literature due to its ability to provide detailed anatomical information, which is crucial for assessing structural changes in the brain associated with AD. The fMRI is the second most utilized modality, accounting for 21% of the studies, reflecting a significant interest in understanding the functional dynamics of the brain in the context of AD. While offering valuable metabolic and neurochemical information, PET-only imaging represented only 3% of the studies; models that integrate sMRI with PET constituted 11% of the studies, highlighting increased interest in multimodal approaches that leverage the complementary strengths of structural and metabolic information. Lastly, despite a clear inter-related modality, the combination of sMRI and fMRI is the least represented modality, representing only 2% of the studies.

### Data sources

This survey investigates papers that utilize data from the Alzheimer's Disease Neuroimaging Initiative (ADNI) database (adni.loni.usc.edu). The ADNI is a longitudinal study that was launched in 2003 as a public–private partnership and its primary goal is to provide data with different modalities including MRI, PET, clinical and neuropsychological assessment specific to progression of AD. The ADNI study now comprises four different cohorts: ADNI1, ADNIGO, ADNI2, and ADNI3. The existing labels of ADNI are AD, cognitive normal control (CN), MCI, and significant memory concern (SMC). The MCI category in the ADNI GO and ADNI2 cohorts is further divided into early MCI (EMCI) and late MCI (LMCI). Moreover, in the evaluation model section, we utilized Open Access Series of Imaging Studies (OASIS) datasets (oasis-brains.org) dataset to assess the generalizability of the ML models.

### Neuroimaging preprocessing methods

Raw neuroimaging data often contain various artifacts and biases that can affect the accuracy and reliability of subsequent analyses. Preprocessing aims to correct these issues and standardize the data across subjects for meaningful comparisons and interpretations. The preprocessing steps for sMRI, fMRI, and PET data may vary slightly based on the specific software and methods used. However, the following are the general preprocessing steps for each of these modalities used by many of the studies.

*Pre-processing of sMRI data* For sMRI data, the process begins with bias field correction to rectify intensity inhomogeneities using methods such as SPM's bias field correction (fil.ion.ucl.ac.uk/spm/). This step is critical for ensuring that the brightness across the image is uniform, allowing a more accurate interpretation of the tissue contrasts. Skull stripping follows a procedure that removes non-brain elements like the scalp and skull, isolating the brain tissue for focused analysis. Image registration is another crucial step involving aligning the structural image to a standardized template, such as the Montreal Neurological Institute (MNI) space, facilitating group analyses. Spatial normalization further processes the image to normalize it to a standard space using transformation matrices computed during registration, allowing for inter-subject comparison. Tissue segmentation partitions the sMRI data into different tissue types, such as gray matter (GM), white matter (WM), and cerebrospinal fluid (CSF), using tools like FSL's FAST (fsl.fmrib.ox.ac.uk/fsl/fslwiki/FAST) or SPM's New Segment. Each segment is vital for different analyses. Finally, intensity normalization normalizes the intensity values across the image to minimize intensity variations caused by scanner differences or acquisition parameters.

*Pre-processing of fMRI data* The preprocessing of fMRI data begins with slice timing correction to correct acquisition time differences between slices of the fMRI sequence using techniques such as interpolation. Motion correction is paramount in fMRI preprocessing, as even slight movements by the subject during the scan can lead to errors. This step aligns all the scan volumes to a reference volume to mitigate these effects. Spatial smoothing is then applied to reduce noise and increase the signal-to-noise ratio using tools like FSL's SUSAN (fsl.fmrib.ox.ac.uk/fsl/fslwiki/SUSAN) or SPM's smoothing. Spatial normalization aligns the fMRI data to a standardized space using the transformation matrix computed during sMRI preprocessing, allowing group analyses and comparison across subjects. Temporal filtering involves applying filters to the data to remove low-frequency drifts (high-pass filtering) and high-frequency noise (low-pass filtering).

*Pre-processing of PET data* PET data preprocessing shares similarities with the previously mentioned modalities, particularly in motion correction and image registration steps. Motion correction for PET aims to correct motion artifacts during the PET scan using tools like SPM’s Realignment. Image registration aligns PET image to sMRI image using intramodal or intermodal registration techniques to improve spatial accuracy and facilitate anatomical reference. In addition, co-registration and intensity normalization are critical steps in PET preprocessing, standardizing the data for subsequent analysis. Co-registration involves mapping the registered PET image onto a standard space using the transformation matrix derived from the co-registered sMRI. Intensity normalization aims to normalize the PET image intensity values to account for differences in scanner calibration and acquisition parameters.

These preprocessing steps help to minimize artifacts, improve image quality, and standardize the data across subjects, thus enabling accurate and reliable analysis and interpretation of neuroimaging data.

### Data analysis strategies

There are four primary strategies for analysis neuroimaging data: voxel-based, slice-based, patch-based, and ROI-based [22, 23], which have been used when developing machine-learning models.

1. *Voxel-based*: The voxel-based method refers to the analysis approaches that operate at the level of individual voxels of data acquired from techniques such as sMRI, fMRI, and PET. This method typically processes full-brain images, capturing the global brain structure, function, or connectivity patterns. The voxel-based analysis allows researchers to examine volumetric or intensity changes in brain regions, investigate functional connectivity between different voxels, or detect abnormalities in specific brain areas. The voxel-based method is widely used in single-modality studies of AD, such as sMRI [15, 24–33] and fMRI [34, 35]. These methods enable researchers to investigate brain structural, functional, and metabolic changes associated with AD by analyzing individual voxels in the images. Additionally, voxel-based method can be applied in multi-modal studies that combine different imaging modalities such as combining sMRI, and PET [36, 37] which may result in comprehensive analysis of AD-related changes.
2. *Slice-based*: Slice-based method refers to an approach where brain image analysis is performed on individual brain slices rather than the entire brain volume. This method involves dividing the three-dimensional (3D) brain volume into two-dimensional (2D) slices, typically using standard projections like *sagittal* (from left to right), *coronal* (from front to back), and *horizontal* (from top to bottom) planes. By extracting 2D slices from 3D brain scans, researchers can analyze them separately. This approach simplifies data management and reduces computational costs for managing and analyzing data. However, slice-based methods and data may lack 3D spatial context and information about the spatial relationship between neighboring slices may be lost. This can be a drawback when studying brain structures or functional connectivity patterns that are distributed in 3D space. The slice-based method is a commonly employed approach in AD ML studies, including studies using sMRI [16–18, 38–53], fMRI [17, 18, 50, 54–56], and PET [57, 58].
3. *ROI-based*: ROIs are specific areas within the brain that the neuroscience community has defined to ensure consistency and standardization in research. Researchers can select specific brain regions or anatomical structures relevant to AD pathology for in-depth analysis. This method allows for increased specificity in the analysis by targeting specific regions of interest within the brain and is particularly advantageous when studying localized brain regions or specific networks. By narrowing down the analysis to relevant ROIs, researchers can gain a more targeted understanding of the functions and connectivity of these regions related to AD. However, the ROI-based approach could potentially lose spatial and functional information and, researchers may miss out on valuable information from surrounding regions that could contribute to a comprehensive understanding of the brain's functioning related to AD. The ROI-based method is also a widely utilized approach in single-modality ML studies of AD, including those involving sMRI [59–61], fMRI[62–73], and PET [74]. Furthermore, ROI-based method can also be applied in multi-modal studies, such as combining sMRI with PET [75], or fMRI [76].
4. *Patch-based*: The patch-based approach is a data management technique that involves dividing the brain image, such as sMRI, fMRI, and PET, into smaller patches or subregions. Depending on the specific analysis, these patches typically have a fixed size and can overlap or be non-overlapping. Instead of analyzing the entire image or voxel-by-voxel analysis, the patch-based approach focuses on extracting features and information from these localized patches. Patch-based analysis is an emerging technique that combines aspects of both voxel, and ROI-based approaches and may strike a balance between capturing local information and maintaining computational efficiency. This approach is particularly well-suited for detecting subtle, localized abnormalities in AD, as it allows for fine-grained analysis while reducing the risk of overfitting. The patch-based method has found its usage in AD studies based on sMRI [77–80], PET [81, 82], sMRI and PET [83–85].

Each data analysis strategy has advantages and limitations, and the choice depends on the AD research objectives and available resources. The second level of Fig. 1 visually shows the percentage of reviewed papers utilizing these four methods across 70 papers. Our analysis reveals that sMRI studies predominantly favor slice-based approach with approx. 44% of studies employing this method. Voxel-based methods are also well-represented in sMRI research at 33%, indicating a significant interest in leveraging the 3D aspects of brain structure. Patch-based and ROI-based methods are less prevalent, each utilized by 11% of the studies.

For fMRI, the data shows a clear inclination towards ROI-based and slice-based approaches, both accounting for 46% of the studies. In contrast, voxel-based methods are considerably less represented in fMRI studies, constituting only 8%, potentially due to the higher computational demands.

In PET imaging, researchers employ an equitable distribution of data analysis strategies, with 25% of papers utilizing each patch-based, ROI-based, slice-based, and voxel-based approach. It is worth noting that when sMRI and PET are combined, around 50% of the studies utilize a patch-based approach to take advantage of the additional information provided by both structural and metabolic data. About 21% of these combined studies use ROI-based methods, while voxel-based methods make up 29%.

Studies that combine sMRI and fMRI data have consistently shown a preference for using ROI-based methods. We are not aware of any studies that have shown the comparative advantages or disadvantages when using other data-management methods.

### Introduction to machine learning and deep learning

Machine learning is a branch of artificial intelligence that enables learning directly from the data [86], with two main categories: supervised learning and unsupervised learning. In supervised learning, the ML algorithm is trained using data that has output labels or target values. The algorithm learns to map input data to output labels by generalizing from the provided examples. Tasks such as classification (assigning labels to data) and regression (predicting numerical values) [87] are common forms of supervised learning models. Unsupervised learning involves training an ML algorithm on an unlabeled dataset without predefined output labels. The objective for such a model is to discover patterns, structures, or relationships within the data [88].

Deep learning (DL) is a subset of machine learning which employs artificial neural networks with multiple layers to model complex patterns within data. Inspired by the human brain's structure, these models learn from the data using hidden layers, and complex patterns of connections and activations [89] For the training of a neural network, Rumelhart et al. [90] introduced backpropagation which minimizes the error between the actual and the desired output by adjusting the weights of the connections in the network in an iterative fashion. The output of a node in a neural network, containing multiple inputs $\left({x}_{1},{x}_{2},\dots ,{x}_{N}\right)$, weights $\left({w}_{1},{w}_{2},\dots ,{w}_{N}\right)$, and bias $\left(b\right)$, is calculated by $f\left({\sum }_{i=1}^{m}{w}_{i}{x}_{i}+b\right)$. In this equation, $f$ is a non-linear activation function [91] allows neural networks to learn non-linear and complex functions. Sigmoid, tangent hyperbolic (tanh) [92], and rectified linear (ReLU) [93] are the main activation functions in neural networks. DL algorithms use these networks to learn and extract patterns and features from large amounts of data, similar to how the brain processes and recognizes patterns [94]. Deep feed-forward networks (FFNs) are the most straightforward kind of deep-neural networks (DNN), where each layer’s node is linked to each following layer’s node [95–97].

#### Traditional machine learning methods for AD diagnosis

Traditional ML refers to a set of algorithms and techniques that have been developed before the rise of DL. These methods are commonly used for various tasks such as classification, regression, and clustering and have been widely used for the diagnosis of AD. Several studies have applied support vector machine (SVM) [62–64, 67, 68, 70–73, 76, 98, 99] and random forest (RF) [71] as traditional ML methods to the classification of AD. These traditional ML methods have shown promise in AD diagnosis, achieving reasonable accuracy in differentiating ADRD patients from healthy individuals. However, MCI and AD have very non-linear boundary conditions which are exacerbated with co-founding factors including related to data acquisition and pre-processing. While these methods have been used in the past, their generalizability with increasing complex data sets are subject to discussion. A brief discussion on the two of the most common traditional ML models are discussed below.

##### Support vector machine

SVMs are supervised learning methods that are commonly used for classification problems. SVMs maximize the margin between hyperplanes of different data types by mapping the input to points in multidimensional space. Multidimensional space is mapped into a higher-dimensional space by a kernel function, such as a Gaussian or polynomial function [100]. Considering SVM’s relatively good performance, SVMs are extensively evaluated in AD classification using neuroimaging data [62–64, 67, 68, 70–73, 76, 98, 99]. For example, Zhang et al. [100] trained a linear SVM classifier with land-mark-based morphological features of sMRI to classify a testing image as CN or AD. In another study, Zhang et al. [72] used a linear SVM for CN and MCI classification based on the multi-view feature learning method with the multi-atlas-based functional connectivity network of rs-fMRI data. Hojjati et al. [64] developed a model based on a sequential features collection algorithm to find optimal subset features of rs-fMRI data and linear SVM for classifying pMCI and sMCI. Zhao et al. [73] used linear SVM for CN and AD classification based on functional connectivity between white matter (WM) and Gray Matter (GM) of rs-fMRI. Chen et al. [62] integrated two linear SVMs for CN and MCI classification based on functional information in both GM and WM. Yu et al. [70] employed a linear SVM for CN and MCI classification based on brain functional networks from rs-fMRI data. SVMs with multi-kernel architectures are more flexible than those with single-kernel architectures. Despite the excellent performance of multi-kernel SVMs, they have much higher computational complexity than single-kernel SVMs. Sadiq et al. [68] proposed a wavelet-based fractal analysis of rs-fMRI brain connectivity for AD classification using linear SVM. Qian et al. [67] employed a nonlinear SVM classifier with a radial basis function (RBF) kernel to accurately identify MCI individuals from CN accurately.

##### Random forest

The Random Forest (RF) is an ensemble algorithm composed of several decision trees as classifiers. A majority voting strategy determines the final output of the RF. It can handle thousands of input variables and is effective on large datasets [101]. However, it is a resource-consuming strategy due to calculating multiple tree branches and merging their outputs. Zhang et al. [71] proposed a two-layer RF approach, with the first layer for feature selection and the second for classification of CN and MCI, combining neuropsychological assessments and rs-fMRI network analysis after feature selection implemented via the RF approach.

#### Deep learning methods for AD diagnosis

Deep Learning has emerged as a promising tool for diagnosing AD, and its utilization in medical applications continues to grow. Different variations in the architectures of deep-learning networks, including convolutional neural networks (CNN), recurrent neural networks (RNN), autoencoders (AE), restricted Boltzmann machines (RBM), deep belief networks (DBN), and vision transformers (ViT), demonstrate the variety of approaches used to tackle the challenge of AD diagnosis (Table 2).

**Table 2:** Literature review of AD diagnosis based on modality, data management, traditional machine learning, deep learning methods, and their accuracy on ADNI dataset

Study	Modality	Data analysis strategy	Classification method	Task	Accuracy (%)
[39]	sMRI	Slice-based	2D CNN	AD vs. CN MCI vs. CN AD vs. MCI	82.8 66 62.5
[42]		Slice-based	2D CNN (ResNet-18) + RNN (LSTM)	CN vs. AD CN vs. MCI	89.5 81.7
[61]		Slice-based	2D CNN (VGGNet-16)	AD vs. CN AD vs. MCI CN vs. MCI AD vs. CN vs. MCI	99.14 99.30 99.22 95.73
[40]		Slice-based	2D CNN (Modified VGGNet-16)	AD vs. CN AD vs. MCI CN vs. MCI AD vs. CN vs. MCI	98.33 93.89 91.67 91.85
[52]		Slice-based	2D CNN (ResNet)	AD vs. CN AD vs. CN vs. MCI	81.3 56.8
[44]		Slice-based	2D CNN (Modified DenseNet-121)	AD vs. CN AD vs. MCI MCI vs. CN	94.97 91.98 74.70
[41]		Slice-based	2D CNN (GoogLeNet, ResNet-18, ResNet-152)	AD vs. MCI vs. LMCI vs. CN	98.88 97.02 99.7
[16]		Slice-based	2D CNN (GoogLeNet, ResNet-18, ResNet-152)	AD vs. MCI vs. LMCI vs. CN	98.88 98.01 98.14
[53]		Slice-based	2D CNN (GoogLeNet, CaffeNet)	CN vs. sMCI vs. pMCI	87.78 83.23
[17]		Slice-based	2D CNN	AD vs. CN AD vs. MCI CN vs. MCI AD vs. CN vs. MCI	99.9 99.7 100 100
[50]		Slice-based	2D CNN (GoogLeNet, LeNet)	AD vs. CN	98.74 97.88
[49]		Slice-based	stacked SAEs and stacked DAEs	AD vs. EMCI vs. LMCI vs. CN	92.44 97.11
[47]		Slice-based	ViT	AD vs. CN	96.8
[43]		Slice-based	ViT	sMCI vs. pMCI	83.27
[46]		Slice-based	ViT	AD vs. CN	88.2
[18]		Slice-based	ViT	AD vs. CN MCI vs. CN AD vs. CN vs. MCI	100 100 87
[79]		Patch-based	2.5D CNN	sMCI vs. pMCI	79.9
[77]		Patch-based	3D CNN	AD vs. CN	86.98
[78]		Patch-based	3D CNN	AD vs. CN MCI vs. CN	89.5 73.8
[80]		Patch-based	stacked DSAE	AD vs. CN MCI vs. CN	88.73 80.91
[81]		Patch-based	3D CNN	AD vs. CN	87.15
[99]		patch-based	SVM	AD vs. CN MCI vs. CN	83.1 73.6
[30]		Voxel-based	SAE and 3D CNN	AD vs. CN AD vs. MCI CN vs. MCI AD vs. CN vs. MCI	95.39 86.84 92.11 89.47
[25]		Voxel-based	3D CNN	AD vs. CN vs. MCI	94.1
[27]	sMRI	Voxel-based	3D CNN	AD vs. CN MCI vs. CN AD vs. CN vs. MCI AD vs. LMCI vs. MCI vs. CN	94 90 87 66
[28]		Voxel-based	3D CNN	AD vs. CN AD vs. EMCI AD vs. LMCI LMCI vs. CN LMCI vs. EMCI EMCI vs. CN	88 66 61 67 47 57
[33]		Voxel-based	3D CNN	AD vs. CN	58.5
[15]		Voxel-based	3D CNN	AD vs. CN	98.74
[29]		Voxel-based	3D CNN + 3D CAEs	AD vs. CN	88.31
[26]		Voxel-based	3D CAE + 3D CNN	AD vs. CN AD vs. MCI MCI vs. CN AD CN vs. MCI	97.6 95 90.8 89.1
[24]		Voxel-based	MLP + RNN (BRGU)	AD vs. CN	89.7
[31]		Voxel-based	3D ConvLSTM	AD vs. CN	86
[124]		Voxel-based	RBM	AD vs. CN	98.6
[60]		ROI-based	Stacked DA	AD vs. CN	94
[61]		ROI-based	DBN + DNN	AD vs. CN MCI vs. CN pMCI vs. sMCI	90.28 74.2 73.28
[59]	sMRI	ROI-based	DBN	AD vs. CN sMCI vs. AD pMCI vs. CN	90 84 83
[98]		ROI-based	SVM	AD vs. CN pMCI vs. sMCI	98.83 80.9
[58]		Slice-based	2D CNN (VGG-16)	AD vs. CN	99.95
[54]		Slice-based	2D CNN (AlexNet)	CN vs. SMC vs. EMCI vs. LMCI vs. AD	97.64
[50]		Slice-based	2D CNN (GoogLeNet, LeNet)	AD vs. CN	94.24 94.32
[55]		Slice-based	2D CNN (LeNet-5)	AD vs. CN	96.85
[17]		Slice-based	2D CNN	AD vs. CN AD vs. MCI CN vs. MCI AD vs. CN vs. MCI	97.5 98.3 97.59 97.43
[56]	fMRI	Slice-based	2D CNN (ResNet-18)	CN vs. SMC vs. EMCI vs. LMCI vs. MCI vs. AD	97.92
[18]		Slice-based	ViT	AD vs. CN MCI vs. CN AD vs. CN vs. MCI	99 97 97
[34]		Voxel-based	3D CNN	AD vs. CN	85.27
[35]		Voxel-based	3D CNN	AD vs. EMCI vs. LMCI vs. CN	93
[65]		ROI-based	Deep AE	MCI vs. CN	87.5
[66]		ROI-based	Deep AE	MCI vs. CN	86.47
[69]		ROI-based	Deep AE	MCI vs. CN	72.58
[68]		ROI-based	SVM	AD vs. CN	83.3
[63]		ROI-based	SVM	AD vs. CN	91.6
[72]		ROI-based	SVM	MCI vs. CN	85.5
[64]		ROI-based	SVM	pMCI vs. sMCI	91.4
[73]		ROI-based	SVM	AD vs. CN	81.11
[62]		ROI-based	SVM	MCI vs. CN	78.7
[70]		ROI-based	SVM	MCI vs. CN	84.8
[67]	fMRI	ROI-based	SVM	MCI vs. CN	93.33
[71]		ROI-based	RF	MCI vs. CN	91.4
[58]	PET	Slice-based	2D CNN (VGG-16)	AD vs. CN	73.46
[128]		Patch-based	3D CNN	AD vs. CN	92.2
[127]		Voxel-based	ViT	AD vs. CN	91.34
[74]		ROI-based	2D CNNs (AlexNets)	Mild MCI vs. Severe MCI	85
[83]	sMRI + PET	Patch-based	CAE + 3D CNN (3D-VGG16)	AD vs. CN AD vs. MCI MCI vs. CN AD vs. MCI vs. CN	98.8 93 95 91.13
[84]		Patch-based	DNN	sMCI vs. pMCI	82.93
[85]		Patch-based	SAE + 3D CNN	AD vs. CN MCI vs. CN	90.3 87.9
[36]		Voxel-based	3D CNN + SBi-RNN	AD vs. CN pMCI vs. CN sMCI vs. CN	94.29 84.66 64.47
[37]		Voxel-based	3D CNN + 2D CNN	AD vs. CN	89.64
[75]		ROI-based	Stacked AEs	AD vs. CN CN vs. MCI CN vs. sMCI vs. pMCI vs. AD	91.4 82.1 53.79
[76]	sMRI + fMRI	ROI-based	SVM	LMCI vs. CN EMCI vs. CN LMCI vs. EMCI	88.5 82.7 79.6

*CNN* Convolutional neural network, *RNN* Recurrent neural network, *DBN* deep belief network, *DNN* Deep neural network, *ViT* Vision transformer, *MLP* Multi-layer perceptron, *SVM* Support vector machine, *RF* Random forest, *AE* Auto-encoder, *SAE* Sparse auto-encoder, *DAE* Denoising auto-encoder, *DSAE* Denoising sparse auto-encoder, *CAE* Convolutional auto-encoder, *SDA* Stacked denoising auto-encoder, *BRGU* Bidirectional gated-recurrent unit, *SBI-RNN* Stacked bidirectional recurrent neural network, *ConvLSTM* Convolutional long short-term memory, *CN* Cognitive normal control, *MCI* Mild cognitive impairment, *EMCI* Early MCI, *LMCI* Late MCI, *sMCI* stable MCI, *pMCI* progressive MCI, *AD* Alzheimer’s Disease, *SMC* Significant memory concern

##### Convolutional neural network (CNN)

LeCun et al. introduced Convolutional Neural Networks (CNN) in 1989 [102], which have proven to be particularly effective due to their ability to leverage spatial information and extract features through stacked convolutional layers. By utilizing convolutional layers, CNNs can learn hierarchical representations of images, where each layer focuses on capturing increasingly complex features [103]. This hierarchical representation allows CNNs to achieve remarkable results in tasks like image classification, object detection, and segmentation [104, 105]. One of the main advantages of CNNs is their ability to combine feature extraction and classification within the same network architecture [103]. This end-to-end learning approach allows CNN to learn discriminative features directly from the raw input data. However, it is important to note that CNNs typically require a large amount of labeled training data to learn effectively, and avoid overfitting [106].

The architecture of a CNN usually includes convolutional layers, activation layers, pooling layers, fully connected layers, and a softmax layer [106]. Several famous CNN architectures have been developed over the years, each with its unique design characteristics and advantages. Some of these architectures include AlexNet [107], VGGNet [108], CaffeNet [109], GoogLeNet [110], DenseNet [111], LeNet [112], Inception [113], and ResNet [114]. Two stages are required to train a CNN: a feed-forward stage and a backward stage. In the feed-forward stage, the loss cost can be calculated using the prediction output along with the ground truth labels. In the backward stage, the chain rules calculate the gradient of each parameter based on the calculated error, and then all parameters are updated based on the gradients. Training (or learning) of CNN can be stopped after sufficient iterations of both stages. The first layer is convolutional, convolving the input image with the learned filters to produce appropriate feature maps. CNNs extract features in their first layers, whereas in their last layers, they use those features to classify tasks [115]. A nonlinear activation function such as a sigmoid, tanh, and ReLU is applied after the convolutional layer to build a feature map for each filter. In this way, models can learn complex representations because of the nonlinear functions. The pooling layer is applied after each convolutional layer, and the feature map is down sampled by applying pooling functions, such as the maximum, minimum, or average. The fully connected layer is implemented after a series of convolutional and pooling layers. A fully connected layer connects features from the previous layer to the output layer. In the final layer, the softmax function is applied for the classification. Below we discuss few of the CNN models that have been used for classification in the ADRD domain.

2D CNN: Many neuroimaging studies have employed 2D CNNs for AD diagnosis [16, 17, 39–42, 44, 45, 50, 52–56, 58, 74]. For example, Aderghal et al. used a 2D CNN with two convolutional layers applied to hippocampus slices of sMRI [39]. Other studies have employed LeNet and GoogLeNet, trained from scratch, for classifying slices obtained from sMRI [51] and fMRI scans [55]. Farooq et al. applied 2D GoogLeNet and ResNet models to sMRI slices [16]. Kazemi and Houghten evaluated the effectiveness of GoogLeNet and AlexNet in classifying different stages of AD using 2D fMRI data [54]. Pre-trained CNNs such as VGG-16 have also been used to classify different stages of AD based on 2D slices of modalities [40, 45]. Some studies have combined 2D CNNs with other architectures. For example, Gao et al. utilized a combination of fine-tuned ResNet-18 and RNN for AD diagnosis [42].

3D CNN: As neuroimaging data inherently has three dimensions, 3D CNNs can capture the spatial relationships between voxels within the volume. This makes them well-suited for analyzing and classifying AD as they can learn spatial patterns and dependencies that may be important for detecting abnormalities or subtle changes associated with the disease. Therefore, as compared to 2D CNNs that only consider individual slices or 2D views of the data, 3D CNNs have gained popularity in many studies for AD diagnosis. Duc et al. [34] and Parmar et al. [35] proposed 3D CNN models with fMRI data. Cui and Liu constructed a 3D CNN-based model to analyze the hippocampus in 3D sMRI [77]. Esmaeilzadeh et al. trained a 3D CNN to classify AD and CN subjects and then used transfer learning to classify subjects into AD, CN, and MCI groups based on sMRI data [25]. Karasawa et al. developed a 3D ResNet-based architecture to prevent feature loss in sMRI data [27]. Some studies have combined 3D CNNs with other architectures to improve the performance of AD diagnosis [36, 81, 85]. Li et al. constructed a model based on 3D CNN and a 3D convolutional autoencoder (CAE) with sMRI data [29]. Payan and Montana designed a hybrid DL framework that applied stacked AEs and 3D CNNs to sMRI data [30]. Moreover, combining 3D CNNs with other architectures has been explored in multi-modality studies to leverage the complementary information provided by different modalities. For instance, Vu et al. combined two 3D CNNs with a stacked AE on fused sMRI and PET [85]. Feng et al. proposed a hybrid model based on 3D CNN and stacked bidirectional RNN for extracting discriminative features from sMRI and PET modalities [36]. Vu et al. designed a hybrid AD classification architecture based on a CAE and 3D-VGG16 applied to sMRI and PET scans [83].

##### Recurrent neural network (RNN)

An RNN is a type of neural network designed to process sequential data. It differs from traditional FFNs because it has an internal memory or hidden state that enables it to retain information about past inputs and use it to make predictions or decisions. The key feature of an RNN is its ability to operate on sequences of data, where each element in the sequence is processed one at a time. The output at each time step depends on the current and previous inputs processed by the network [116, 117]. An RNN combines an input with the hidden state from the previous time step to produce an output at each time step. The hidden state serves as a memory of the past inputs and helps capture temporal dependencies in the sequence. The output can be used for making predictions, and the hidden state is updated and passed on to the next time step. However, one limitation of traditional RNNs is their tendency to suffer from the vanishing or exploding gradient problem, leading to difficulties in learning long-term dependencies. To overcome this issue, more advanced forms of RNNs have been developed, such as Long Short-Term Memory (LSTM) and Gated Recurrent Unit (GRU) [116]. These variants use specialized memory cells and gating mechanisms to capture long-term dependencies better and alleviate the vanishing gradient problem.

RNNs have been applied in neuroimaging studies for AD diagnosis [24, 36, 42]. Feng et al., multiple 3D CNNs are applied to each modality of the input data. It helps capture detailed information from each modality. Then, bidirectional RNNs are stacked at the end to obtain more comprehensive and contextual information from the sequential input data [36]. In the architecture designed by Cui et al., two stacked GRU layers are used for longitudinal feature learning. The GRU layers help overcome the short-term memory problem in RNNs, and retain information from previous iterations. This architecture effectively captures temporal dependencies in the input data [24]. Gao et al. developed an architecture based on LSTM to extract longitudinal features that could effectively capture and retain long-term dependencies in the input data [42].

##### Auto-encoder (AE)

The AE is a type of neural network architecture that consists of two main components: an encoder and a decoder. An AE aims to learn an efficient representation or encoding of the input data, typically by compressing it into a lower-dimensional latent space. This compressed representation is then decoded back to the original input space [118, 119].

During the training process, the AE aims to minimize the reconstruction error, which measures how well the decoder can reconstruct the original input from the encoded representation [118]. By doing so, the AE learns to capture the most important features or patterns in the data while discarding irrelevant or redundant information. Several variations of AEs have been used in different studies. Stacked AEs are created by stacking multiple layers of encoders and decoders together. Each layer in the stack takes the encoded representation from the previous layer as input and further compresses it [118, 119]. It allows for discovering more complex and nonlinear patterns in the data. Sparse AE (SAEs) introduces sparsity constraints during training, encouraging the model to learn sparse representations [118]. Denoising AE (DAE) adds noise to the input data during training, forcing the model to learn robust representations that can handle noisy inputs [118, 120]. Convolutional AE (CAE) utilizes convolutional layers, which are particularly effective for processing spatial data such as images [118, 121].

In various AD research studies, stacked SAEs with two [65, 66, 75, 122] or three [82, 84] hidden layers and a softmax layer have been employed for both single-modality [65, 66, 82] and multi-modality [75, 84, 122] data. In the study by Shi et al., they proposed a model based on stacked denoising SAEs, allowing for the extraction of hierarchical and abstract features from the sMRI data [80]. In another study, Moussavi-Khalkhali et al. utilized an architecture based on SAEs and DAEs consisting of three hidden layers and a softmax layer [49]. The SAEs and DAEs were stacked together, allowing the model to learn representations of the sMRI data with increased depth and complexity. Integrating DAEs and SAEs can provide complementary benefits in denoising the input and learning more robust and informative representations.

##### Restricted Boltzmann machine (RBM)

In 2007, Hinton [123] proposed the RBM algorithm, an undirected generative stochastic neural network consisting of two layers: visible and hidden. These two layers are connected symmetrically, meaning no intralayer connections are within the visible or hidden layers. The visible units represent the input data, while the hidden units capture higher-level abstract representations or features learned from the input data. The connections between the visible and hidden units are weighted, and the weights are adjusted during the training process using a technique known as contrastive divergence or other gradient-based optimization algorithms. The RBM training process involves two main steps: the forward and the backward pass. In the forward pass, the visible units are used to calculate the activations of the hidden units. In the backward pass, the activations of the hidden units are used to reconstruct the activations of the visible units. The difference between the original and the reconstructed visible units is used to update the weights to minimize the reconstruction error. Building upon RBMs, Singh and Junghel [124] developed a model using three layers of RBMs based on sMRI data for early detection and classification of AD. Ortiz et al. [59] applied an ensemble of DBNs, constructed by stacking multiple layers of RBMs, to all ROIs of sMRI data, and the final prediction was determined using a voting strategy.

##### Vision transformer

**Fig. 2:**
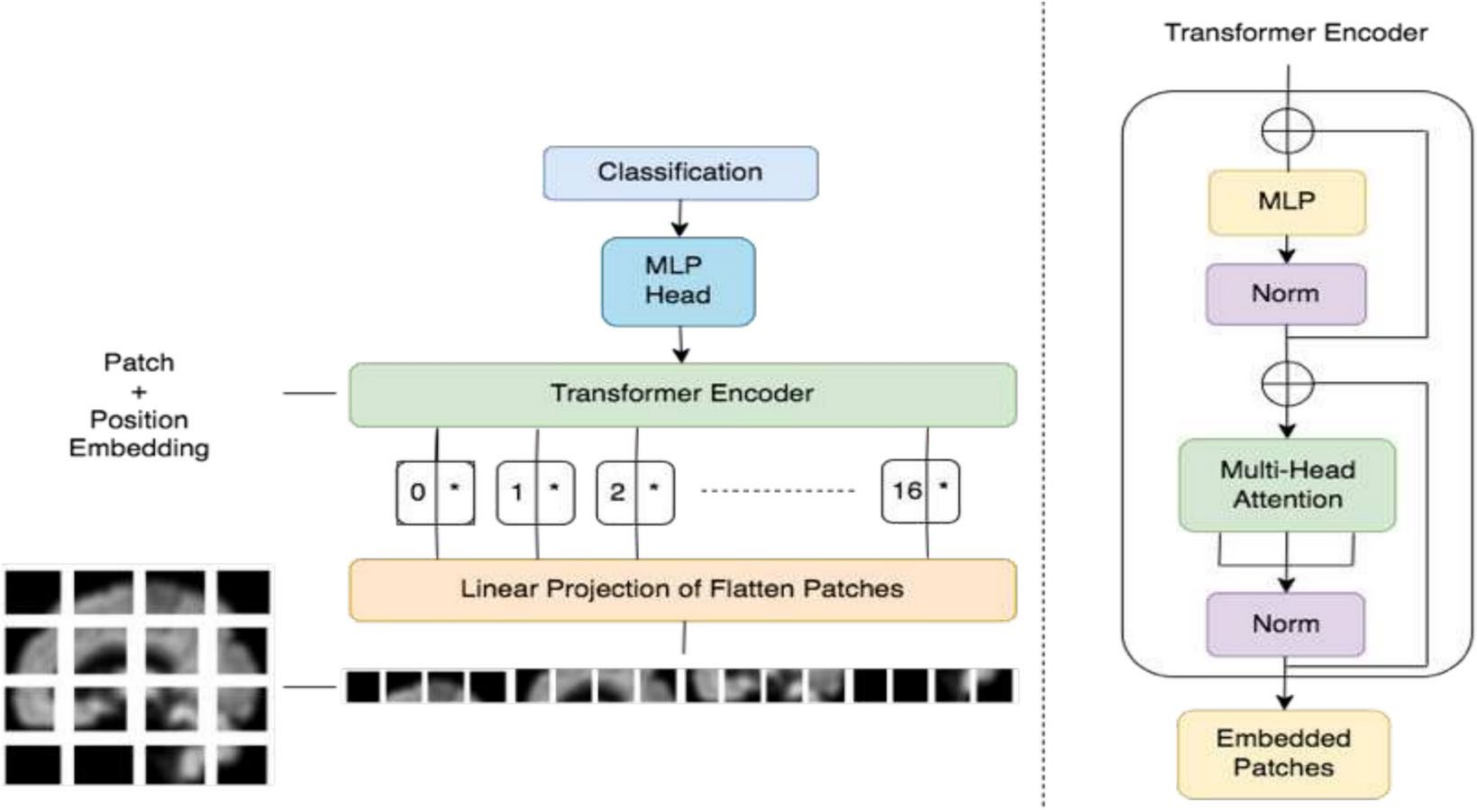
Transformer architecture is shown to process MRI imaging data. Transformers process sequential data using self-attention mechanisms that allow them to weigh the importance of different parts of the input sequence, enabling efficient learning of dependencies and relationships between elements

The Transformer model, introduced by Vaswani et al. [125], is a DL model with an encoder-decoder structure. It has been widely used in various natural language processing tasks. The Vision Transformer (ViT), proposed by Dosovitskiy et al. [126], is a variant of the Transformer model specifically designed for image classification tasks. It only utilizes the encoder part of the Transformer model. The ViT consists of a stack of self-attention mechanisms, a normalization layer, and an FFN. Figure 2 shows the architecture of ViT. First, it divides a given image into patches and then flattens each patch into a vector. These patch vectors are then linearly projected, and a learnable positional embedding is added. Finally, a class embedding vector is concatenated with the patch vectors and fed into the Transformer encoder.

The use of ViTs in AD research has gained attention in recent years. Sarraf et al. [18] proposed an extension of the ViT model called OViTAD, which utilizes both sMRI and fMRI data to predict different stages of AD. They reported that the OViTAD model outperformed CNN-based models in their experiments. Lyu et al. [47] also explored the use of ViT for AD classification. They proposed a cross-domain transfer learning method using ViT and achieved comparable classification performance to recent studies. Hoang et al. [43] applied the ViT model to mid-sagittal sMRI data to predict the conversion from MCI to AD. They found that the ViT model improved the prediction accuracy compared to other techniques. Kushol et al. [46] presented a fusion transformer model called Addformer for AD detection using selected coronal 2D slices of sMRI data. Their experimental results showed that Addformer outperformed traditional methods in terms of classification accuracy. Xing et al. [127] developed a new ViT model called Advit for AD diagnosis using multi-modality PET scans. They reported that Advit achieved better performance than 3D CNN baseline models.

## Comparative analysis of deep learning models for AD diagnosis

The comparative analysis of models that are available for classification can be categorized into three parts. While there is no way to compare fairly among different models, these categorizations enable us to learn emphasis, or lack thereof, for many of the modalities, models, and classes.

### Categorization based on data-modality

The third level of Fig. 1 visually represents the percentage of reviewed papers that have used traditional ML and DL methods for diagnosing AD across 70 papers.

*sMRI studies* According to this figure, in AD-reviewed papers, particularly within sMRI studies, there is a notable tendency to rely on 2D CNNs for slice-based methods, accounting for about 77% of the studies. When it comes to voxel-based approach, 3D CNNs likely take precedence, potentially being used in around 46% of the cases, leveraging their capability to handle the 3D spatial context. On the other hand, about one-third of sMRI studies that use the ROI-based method tend to use DBN, DBN + DNN, and SVM. Studies involving sMRI and patch-based methods adopted DL and traditional ML methods, such as 3D CNN and SVM, accounting for about 44% and 22%, respectively.

*fMRI studies* Slice-based fMRI studies are likely to favor 2D CNNs (about 75%). On the other hand, 3D CNNs are used in all voxel-based fMRI studies. Regarding ROI-based methods in fMRI, SVMs are the most popular choice, used in around 67% of studies. In PET imaging, 2D CNN, 3D CNN, and ViT are equally favored classification methods across all data analysis strategies. When researchers combine different modalities like sMRI and PET, it becomes challenging to classify and analyze the data. In such cases, complex neural network architectures or ensemble methods are used in about 80% of the studies. These methods are chosen to integrate and leverage each modality's strengths effectively.

*sMRI + fMRI studies* In studies that use sMRI and fMRI data, the ROI-based methods reveal a unanimous preference for SVM. Overall, across all these studies, there has been a shift towards more advanced ML approaches in AD research. However, traditional ML methods such as SVM are still significant depending on the specific data analysis strategy and modality. Using a combination of techniques highlights the development of more comprehensive and nuanced diagnostic ML models in the study of AD.

### Categorization based on ML model type

Table 2 and Fig. 1 provides detailed information on the literature review of AD diagnosis, including modalities, data analysis strategies, and traditional ML and DL methods for this survey.

*2D models* Aderghal et al. [39], Jain et al. [45], and Billones et al. [40] predominantly used slice-based methods with 2D CNNs. These studies leverage the strong feature extraction capabilities of CNNs on individual sMRI slices to classify between AD, MCI, and CN. An advantage of this approach is the CNN's ability to capture intricate patterns in 2D slices, which is critical for identifying the subtle changes associated with AD. However, one limitation is the potential loss of spatial context when considering slices individually rather than as part of a whole 3D structure.

*3D models* Some studies, such as Cui and Liu [77] and Payan and Montana [30], utilized patch-based and voxel-based approaches with 3D CNNs. These methods consider the 3D nature of brain images, preserving spatial relationships that might be crucial for AD diagnosis. However, these methods can be computationally expensive and may require significant data preprocessing to manage the higher dimensionality of the input data.

*Hybrid models* Some studies used hybrid models, which combine different neural network architectures like CNNs with AEs, aiming to leverage the strengths of each approach. These models can provide a rich feature set for classification tasks. However, the complexity of these models may lead to overfitting, especially in the case of limited training data typical of medical imaging datasets. For example, Gao et al. [42] introduced a novel combination of CNNs with RNNs, specifically LSTM units, to capture temporal dependencies across slices. This method could recognize patterns over sequential slices, offering a more comprehensive analysis than single-slice methods. While this provides a richer representation of the brain's structure, the increased model complexity can lead to longer training times and require more data for optimal performance.

*Transformer models* Recent studies by Lyu et al. [47], Sarraf et al. [18], and Xing et al. [127] have adopted ViTs for AD classification. ViTs represent a significant shift from traditional CNNs, as they treat the image as a sequence of patches and can capture long-range dependencies between them. This approach has the advantage of focusing on global information, which could be particularly beneficial in capturing the widespread effects of AD on brain structure. Nonetheless, ViTs are data-hungry models that require substantial computational resources, making them less accessible for smaller research settings.

### Categorization based on classification class

The heterogeneity of ADRD diagnosis, and a lack of standard definition of various longitudinal stages of ADRD lends multiple classes to various benchmarks available to computational scientists. These benchmarks and their categorization, for most part, leads to various classes that are used by different models. Since different classes are used in models, it is difficult to compare the results since some classes are easier to classify than others e.g. it is “easier” to classify between healthy control and AD classes, as compared to stable MCI (sMCI) and progressive MCI (pMCI). Nevertheless, presenting data for different models with different classes will enable us to understand the current landscape of ML models and their effectiveness.

**Fig. 3:**
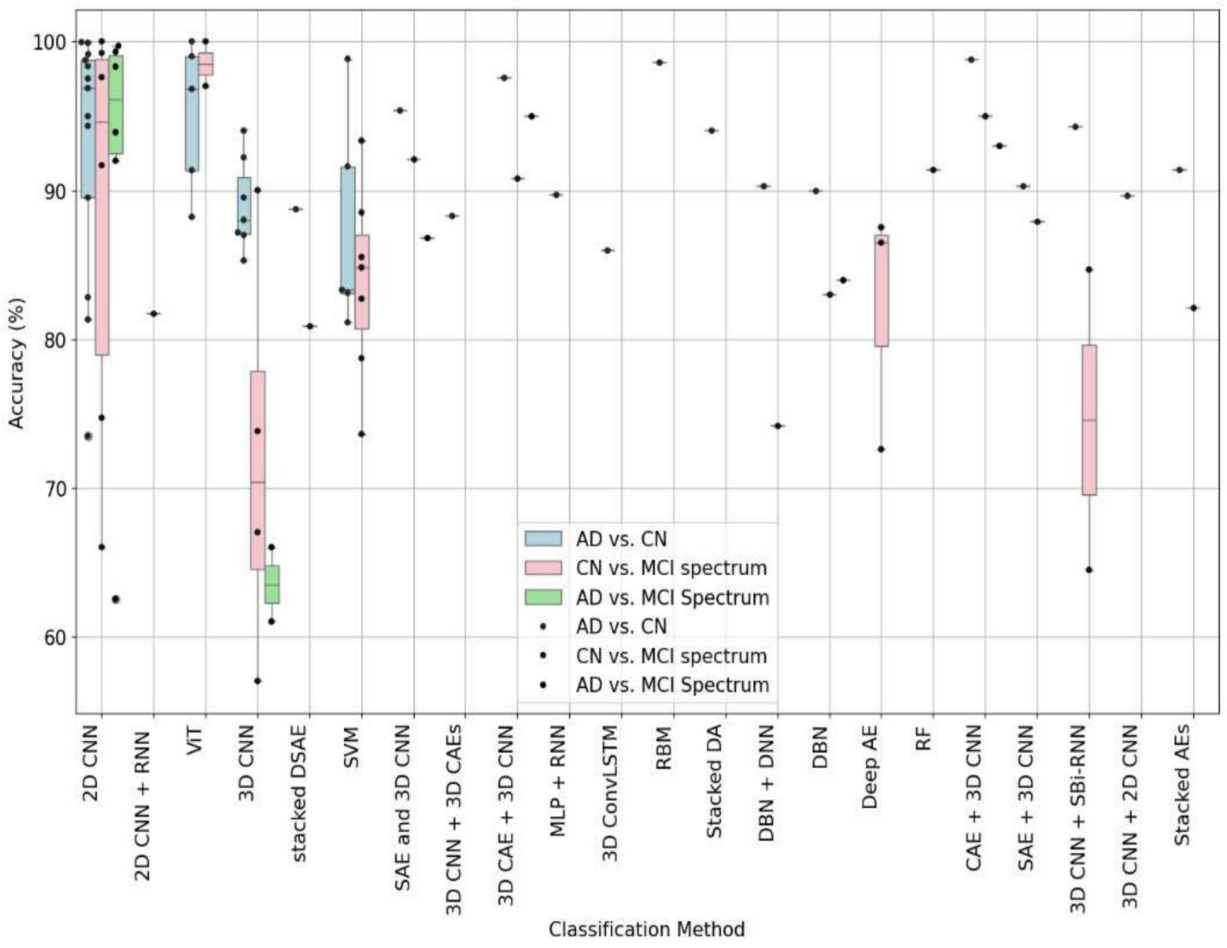
Classification methods and their associated accuracy for binary classification tasks for various classification methods across different binary tasks related to AD, CN, and MCI spectrum (sMCI, pMCI, MCI, EMCI, LMCI). Each box plot displays the median accuracy as a line within the box, the interquartile range (IQR) as the box itself, and the range excluding outliers by the lines or whiskers that extend from the box. Individual points outside the whiskers indicate outliers

Figure 3 comprehensively compares the accuracy of various classification methods across different binary tasks related to AD, CN, and MCI spectrum (sMCI, pMCI, MCI, EMCI, LMCI). Each box plot displays the median accuracy as a line within the box, the interquartile range (IQR) as the box itself, and the range excluding outliers by the lines or whiskers that extend from the box. Individual points outside the whiskers indicate outliers. This figure shows that methods like 2D CNN and ViT demonstrate high median accuracies for distinguishing between AD and CN, with a relatively compact IQR, suggesting consistent results. In differentiating CN from the MCI spectrum, the 2D CNN and ViT methods stand out with a high median accuracy, albeit accompanied by a broad range and lower outliers, pointing to occasional performance dips. Similarly, classifying the AD vs. MCI spectrum reveals high median accuracies. Notably, the 3D CNN method has a lower median accuracy and a more extensive spread in results for this task, highlighting challenges in distinguishing between AD and MCI spectrum conditions.

**Fig. 4:**
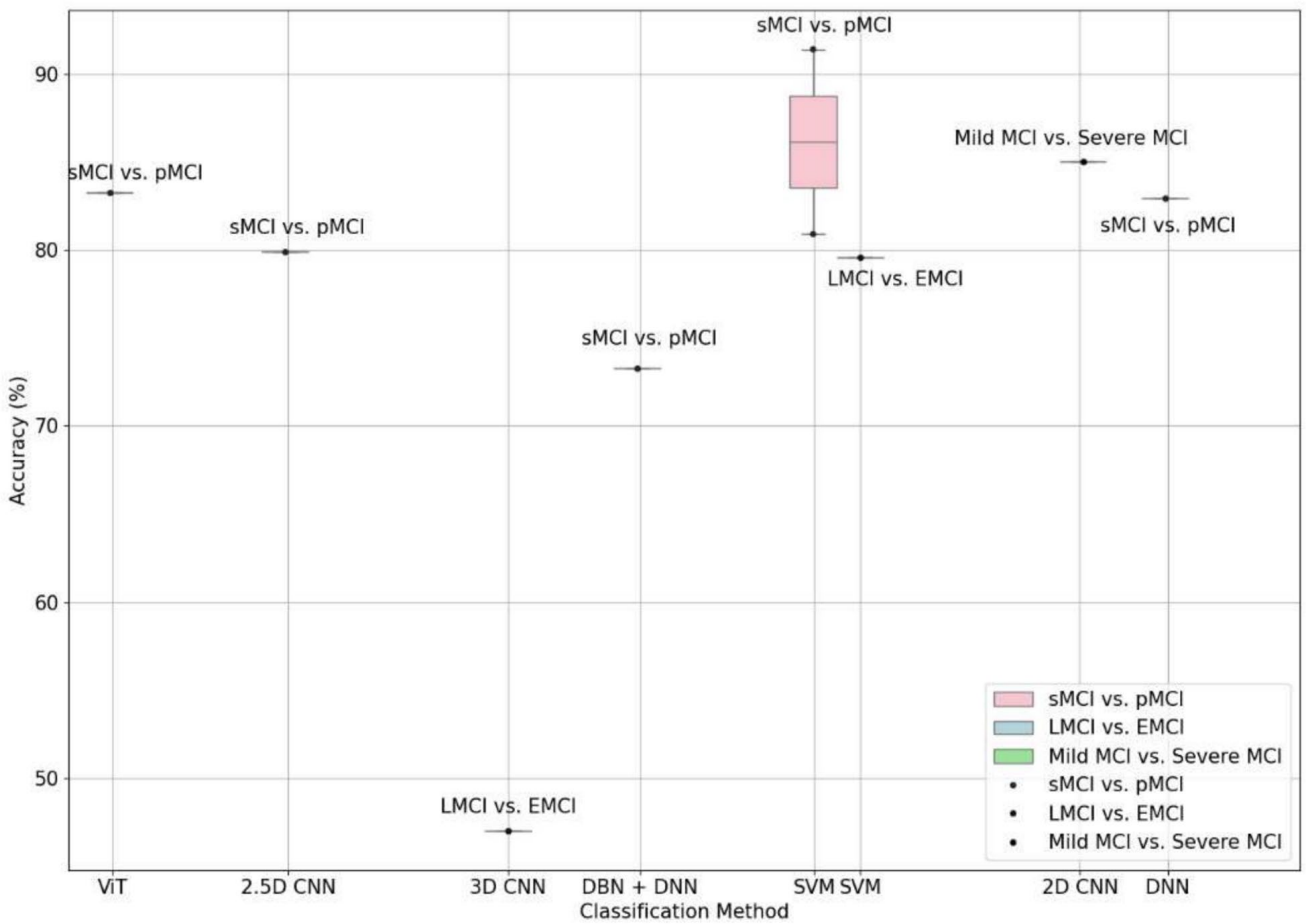
Classification methods and their associated accuracy for binary MCI spectrum tasks for all the studies that were analyzed for this paper. As can be seen in the figure that there are only some papers on classifying between sMCI and pMCI, with some variation in the results for SVM based methods. Deep learning models have not been sufficiently investigated

Figure 4 focuses on comparing the binary classification accuracy for differentiating various stages of MCI (sMCI vs. pMCI, LMCI vs. EMCI, and Mild MCI vs. Severe MCI) using various models. The accuracy percentages span from just above 45% to approximately 90%. For sMCI and pMCI classification, the box for the SVM classification method shows a median accuracy of around 85%, with the lower quartile close to 82% and the upper quartile around 88%. Other classification methods show only individual data points, indicating only a small number of models for such a classification highlighting challenges in distinguishing between MCI spectrum conditions. These points indicate wide variability in accuracy among the methods, with most achieving around 70% to 90%.

**Fig. 5:**
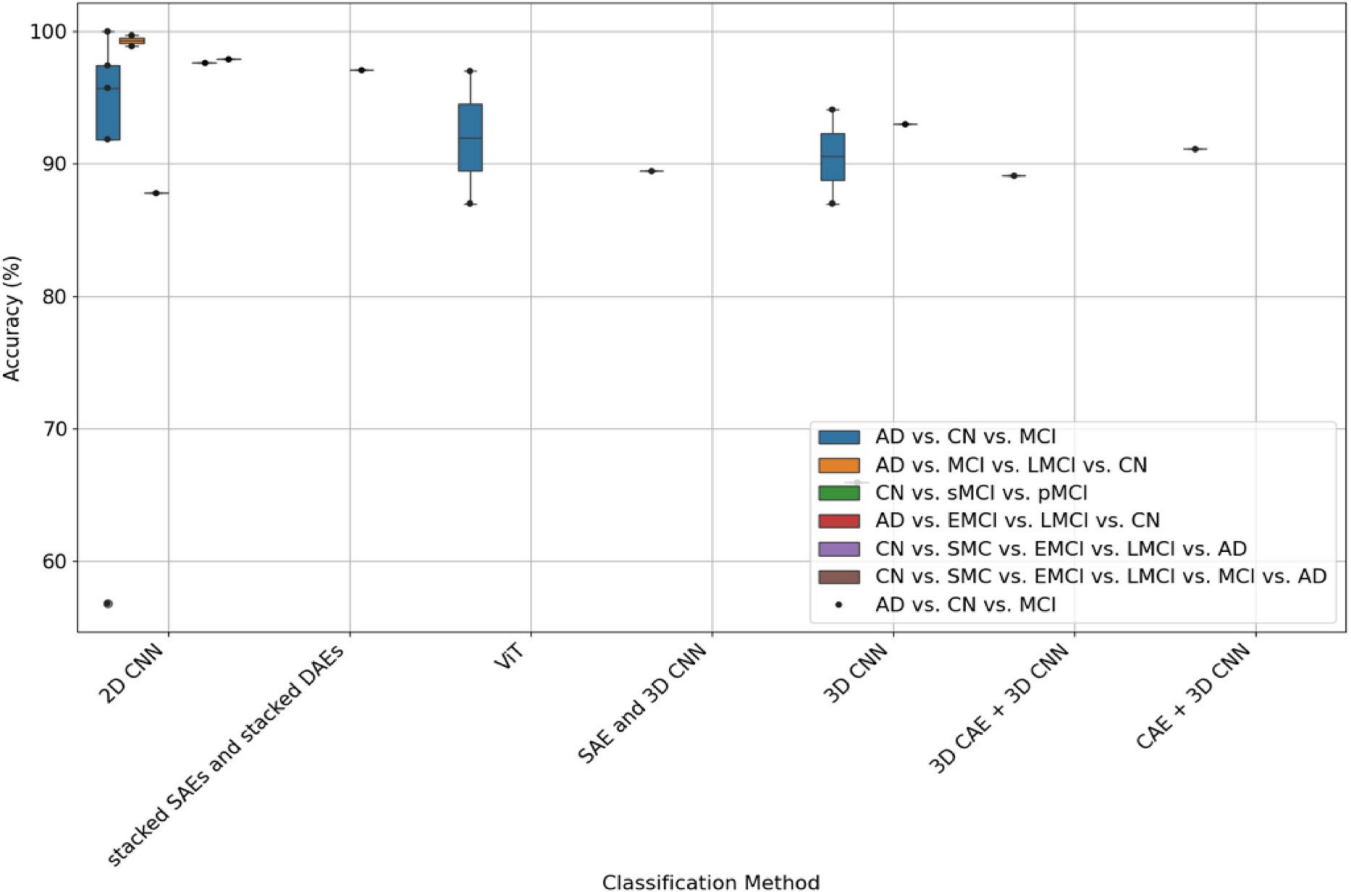
Classification methods and their associated accuracy for multi-label classification tasks (AD vs. CN vs. MCI, CN vs. sMCI vs. pMCI, AD vs. MCI vs. LMCI vs. CN, AD vs. EMCI vs. LMCI vs. CN, CN vs. SMC vs. EMCI vs. LMCI vs. AD, and CN vs. SMC vs. EMCI vs. LMCI vs. MCI vs. AD). In addition to significant variation in how different classes are used by ML models, there is significant variation in the reported accuracy for these models

Figure 5 presents a comparison of the accuracy of various classification methods for distinguishing multi-label classification tasks (AD vs. CN vs. MCI, CN vs. sMCI vs. pMCI, AD vs. MCI vs. LMCI vs. CN, AD vs. EMCI vs. LMCI vs. CN, CN vs. SMC vs. EMCI vs. LMCI vs. AD, and CN vs. SMC vs. EMCI vs. LMCI vs. MCI vs. AD) based on the papers reviewed in this survey. The box plot reveals a substantial variation in the performance of different classification methods, spanning from the lower 60 s to 100%, considering the multi-label nature of the tasks. The 2D CNN method displays a wide range of accuracy percentages, with several points lying far outside the box, denoting a less consistent performance. However, the ViT and 3D CNN methods showcase higher median accuracies with smaller variation in the results indicating further investigation is warranted.

## Evaluating the reproducibility and generalizability of ML models

**Fig. 6:**
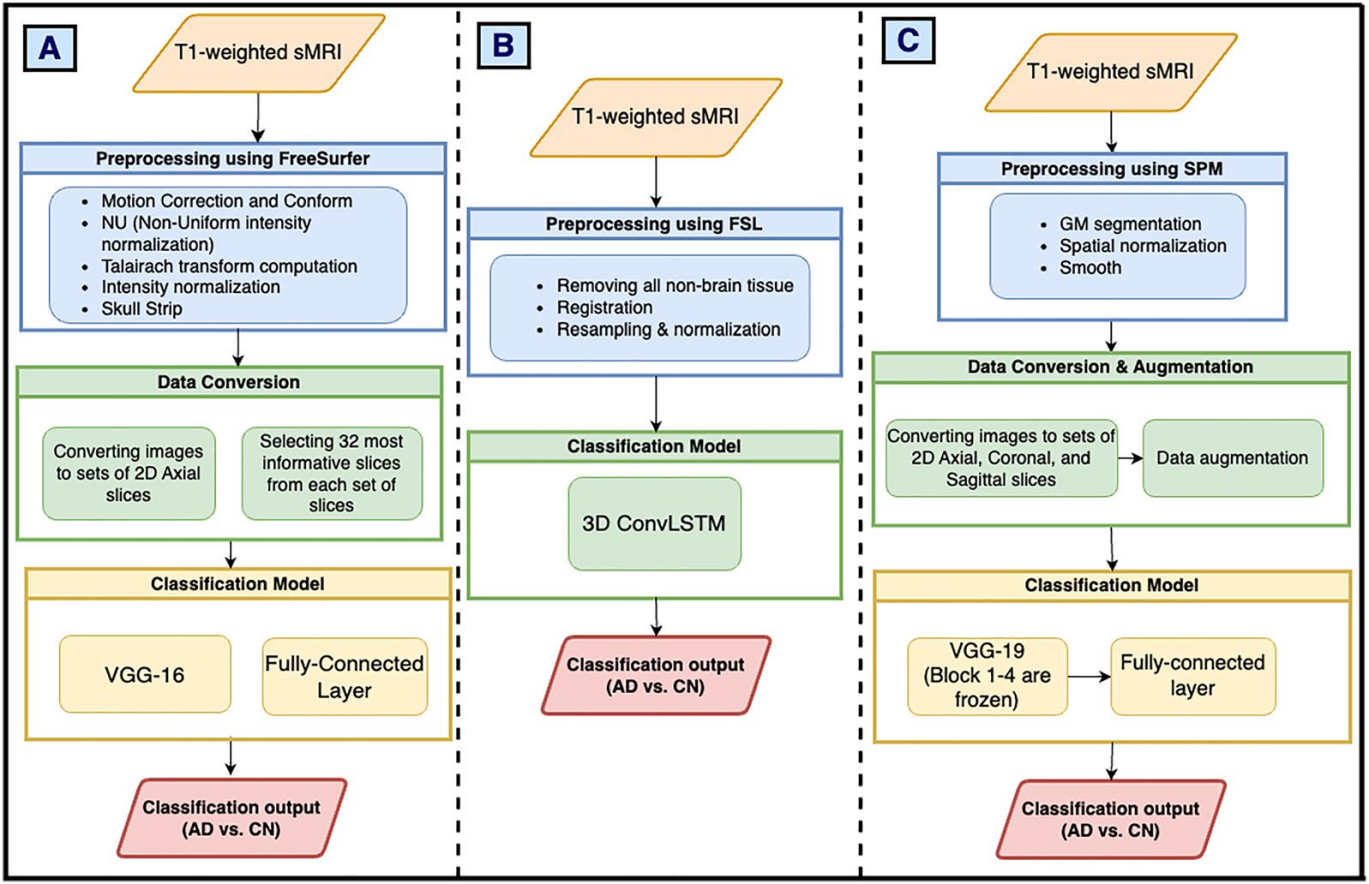
Evaluation workflow followed for the following studies: **A** Jain et al. [45], **B** Tomassini et al. [31], **C** Mehmood et al. [48]. The evaluation workflow enables us to investigate both the reproducibility and generalizability of the models for a given data set

In this section, we will evaluate the reproducibility and generalizability of ML models that are available as open-source, namely Jain et al. [45], Tomassini et al. [31], and Mehmood et al. [48]. Reproducibility of the model will be evaluated by using the same data set that was reported in their study, while generalizability will be studied by evaluating the model on a different cohort of data. Figure 6 shows the evaluation workflow for our paper.

Evaluating reproducibility and generalizability is crucial for ensuring the reliability and broader applicability of ML models in clinical settings. Reproducibility establishes whether the models’ performance can be replicated under the same experimental conditions, while generalizability assesses their ability to handle unseen data with different characteristics. Together, these evaluations highlight the robustness of ML models and their potential for real-world deployment.

### Datasets and preprocessing methods

The evaluation of reproducibility and generalizability was conducted using ADNI and OASIS datasets. Both datasets consist of T1-weighted sMRI data used to distinguish AD patients from CN subjects.

*Ensuring consistency in data selection and experimentation* Ensuring consistency in data selection and preparation is critical for achieving reliable and comparable results in ML studies. In this work, we aligned our experiments with prior research by using standardized datasets, specifically ADNI and OASIS, which are widely recognized in AD research. To ensure fair evaluation, we pre-processed data using the same pipeline reported in the original study: Jain et al. [45] using FreeSurfer software for motion correction, intensity normalization, Talairach transformation, and skull stripping; Tomassini et al. [31] employed FSL for non-brain tissue removal, registration, and normalization; Mehmood et al. [48] used SPM for GM segmentation, spatial normalization, and smoothing. This ensures that we minimize variability introduced by preprocessing differences. Additionally, we adhered to the same data splits used in the original studies, such as 80:20 or 60:20:20 splits for training, validation, and testing, ensuring fair comparisons. The experiments were run multiple times to ensure that the results were consistent, and average of the results were reported. To evaluate generalizability, we conducted cross-dataset validation by training models on one dataset (e.g., ADNI) and testing on another (e.g., OASIS). This approach allowed us to assess the robustness of models across diverse cohorts and imaging protocols. All experimental details, including preprocessing steps, hyperparameters, and software versions, were meticulously documented to ensure reproducibility and transparency.

### Methods

Each of the open-source studies introduced a distinct classification model. Jain et al. [45] utilized a VGG-16 architecture with a fully connected layer for classification, while Tomassini et al. [31] adopted a 3D ConvLSTM model. Mehmood et al. [48] used a VGG-19 model with specific layers frozen during training to leverage pre-learned features. The differences in these architectures provided a diverse perspective on reproducibility and generalizability within the context of AD diagnosis.

### Evaluating the robustness of the existing models reproducibility and generalizability assessment

#### Reproducibility assessment

All three models have reported results for ADNI data sets. Therefore, we re-executed experiments using the same preprocessing pipeline and ADNI data sets for all three models. The reproducibility analysis revealed significant variations among the models when tested using the same experimental conditions and datasets. Jain et al. [45] demonstrated strong reproducibility, achieving an accuracy of 97.96% compared to the reported 99.14%. This high level of reproducibility underscores the robustness of Jain et al.'s model.

In contrast, the models by Tomassini et al. [31] and Mehmood et al. [48] showed significant gaps in reproducibility. Tomassini et al.'s reproduced accuracy of 58% was markedly lower than the reported 86%, indicating potential overfitting in the original study or the omission of critical details in their methodology. Similarly, Mehmood et al.’s reproduced accuracy of 74.2% fell far short of the reported 98.7%, suggesting that the original results may have been influenced by specific experimental conditions that were not explicitly disclosed. These findings highlight the importance of comprehensive reporting in ML studies, including detailed descriptions of preprocessing steps, hyperparameter settings, and data handling, to enable accurate replication.

#### Generalizability assessment

Generalizability, which assesses a model's ability to perform well on unseen data from different distributions, is equally critical for real-world deployment. The generalizability was evaluated by cross testing the models using the ADNI and OASIS datasets. The evaluation using cross-dataset testing between ADNI and OASIS datasets revealed stark differences in the generalization capabilities of the models. Table 3 shows each model's reproduced and generalized classification performance based on ADNI and OASIS datasets.

**Table 3:** Reproduced and generalized classification performance in ADNI and OASIS datasets using tools that were available as open source

Study	Preprocessing tool		Method	Train data	Test data	Accuracy (%)			Test type	Task
	Original study	This paper				Original study	Our experiments			
							Reproduced results	Generalized Results		
Tomassini et al. [31]	FSL	FSL	3D sMRI + ConvLSTM	ADNI	ADNI	–	–	74	Train:Val: Test (60:20:20)	AD vs CN
					OASIS	–	–	50		
				OASIS	ADNI	–	–	48		
					OASIS	–	–	49		
				ADNI + OASIS	ADNI + OASIS	86	58	–		
Jain et al.[45]	Free-Surfer	Free-Surfer	2D Slices + 2D CNN	ADNI	ADNI	99.14	97.96	–	Train:Test (80:20)	
					OASIS	–	–	66.87		
				OASIS	ADNI	–	–	67.96		
					OASIS	–	–	95.78		
Mehmood et al. [48]	SPM	SPM	2D Slices + 2D CNN	ADNI	ADNI	98.73	74.19	–	Train:Test (80:20)	
					OASIS	–	–	78.33		
				OASIS	ADNI	–	–	64		
					OASIS	–	–	71.66		

*Expt* Experiments, *CN* Cognitive normal control, *AD* Alzheimer’s Disease

When Tomassini et al.’s [31] model was subjected to a generalizability study, significant drop in accuracy was observed both when trained and tested within the same data group (ADNI training/ADNI testing (74%), OASIS training/OASIS testing(49%)), and when tested outside the same data-group (ADNI training/OASIS testing (50%), OASIS training/ADNI testing (48%)). When doing the experimentation, it seemed that when tested within the same group, the model seemed to perform well, but this did not extend when another data set was used to do the same experiment. Therefore, we conclude that this model did not generalize well, especially when tested in the out-of-distribution data. These results suggest that the model overfitted to its training data and struggled to adapt to out-of-distribution samples. This lack of robustness limits its applicability in clinical scenarios, where patient demographics and imaging protocols often vary.

When Jain et al. [45] model was subjected to generalizability protocol, the model demonstrated better generalizability though significant drops in the numbers were observed. When trained and tested within the same data group (ADNI training/ADNI testing (97%), OASIS training/OASIS testing (95.7%)), and when tested outside the same data group (ADNI training/OASIS testing (66.8%), OASIS training/ADNI testing (67.9%)) were observed. These numbers suggest high within-dataset accuracies, while the reduction in cross-dataset accuracies suggests that the model is prone to data bias but may generalize well for the same population or demographics subset. The higher numbers are also consistent with the high reproducibility numbers observed for this model [45]. These results align with the high reproducibility of Jain et al.'s model, reinforcing its potential for broader clinical use.

When Mehmood et al.’s [48] model was subjected to the generalizability protocol, we observed mixed results. When trained and tested within the same data group (ADNI training/ADNI testing (74.2%), OASIS training/OASIS testing (71.6%)), and when tested cross-data group (ADNI training/OASIS testing (78.3%), OASIS training/ADNI testing (64%)) were observed. While the model does drop in numbers when tested, we observe that the numbers we get from the generalizability protocol are within a margin of error compared to the numbers we got when trying to reproduce these results. This suggests that the model [48], while it does not exhibit very high accuracy numbers, performs well when tested against other data cohorts, and the changes in the accuracy number may not be statistically significant, suggesting the robustness of the model.

#### Implications

The results of this evaluation underscore several challenges in developing reproducible and generalizable ML models for AD diagnosis. High reproducibility does not always translate to high generalizability, as seen in Jain et al. [45], where strong reproducibility was accompanied by moderate performance on out-of-distribution data. This highlights the need for models to not only replicate well under controlled conditions but also adapt to diverse and unseen datasets. The poor generalizability of Tomassini et al. [31] and the mixed results from Mehmood et al. [48] suggest that dataset-specific biases and overfitting remain significant obstacles. These issues are exacerbated by the lack of diversity in training datasets, which may not capture the full range of variability in real-world clinical data. Cross-dataset validation, as performed in this study, is a critical step for identifying and addressing these biases, ensuring that models are robust and reliable across different populations and imaging protocols. In addition, it is not yet clear what signature these ML models are learning since variability in imaging protocols, as well as site-specific changes (e.g. different MRI machines) can results in models learn different signatures that may be specific to the site but not necessarily the biological or neurological markers that the models are intended to learn. Computational scientists must work out the details to devise methods that can differentiate between site-specific protocols and neurological signatures.

## Discussion

Given that the brain changes due to AD prior to any symptoms, brain imaging intrinsically has the data that can distinguish between AD, progressive MCI and stable MCI. Comparison across literature indicates increased average accuracy for (AD vs. HC) or (MCI vs. HC) approaches for single modality (88.4%) and multiple modality (87.5%) when compared to the methods that classify between pMCI vs. sMCI (77.3% for single modality and 81.3% for multiple modalities). Further, average accuracy for DL models (87.9%) across all modalities relative to traditional ML models (85.5%) revealed modest gains. However, significant research gaps were observed for pMCI vs. sMCI classification using fMRI + sMRI data despite overwhelming evidence that multiple modalities may reveal better classification. Furthermore, DL models for pMCI vs. sMCI using only sMRI data have an average accuracy of 84.5%, indicating that fMRI integration may be useful, and further formal evaluation is warranted. In addition, models that can classify between HC, MCI, and AD (and their variants) seem to perform better for single modalities (90.7%) as compared to multiple modalities (72.5%), indicating that more systematic and formal investigations are needed to design and develop multimodal models. While these are all reported numbers, the reproducibility and generalizability of these models were systematically investigated, which leads us to believe that more research is warranted to ensure the reliability and robustness of these models that can reach clinical practice.

The technical hurdle of using a limited labeled data set (Table 3), which may not traverse the feature search space, results in the subpar performance of these models with data distribution shift, contributing to the ongoing reproducibility crisis. Overall, our extensive experimentation with models available as open-source code and prior literature demonstrates limited accuracy and generalizability in assessing biomarkers using current ML methods, especially when unknown data (from other races or other cohorts) is fed to these ML models (Table 3). Consequently, the existing ML models increase, rather than decrease, the biases in diagnostic criteria, leading to an increase in health disparities related to AD/ADRD linked to environmental, sociocultural, behavioral, and biological factors.

In scouting through the literature related to ML models for ADRD diagnosis and classification, we have identified two separate but related challenges that must be addressed for better understanding and utilization of these models.

One of the primary challenges researchers faces is heterogeneity, both in how data is collected and how it is interpreted. For the collection side, the heterogeneity is expressed in terms of scanner types, imaging parameters, and clinical protocols used. On the interpretation side, heterogeneity is introduced due to varying clinical diagnostic criteria that are used to classify patients at different stages of ADRD. Non-uniformity in the diagnostic criteria leads to non-uniformity in the class labels that are used to express the imaging data. Since class labels are non-uniform, the training and learning from the models remain inconsistent at best and non-usable at worst. There is a wide variation in classification classes, ranging from binary classification (e.g., AD vs. CN) to multi-class classification (e.g., AD vs. CN vs. MCI vs. LMCI), increasing the complexity of the task and requiring more robust and generalized models. Other factors, such as demographics, age, gender, and pre-processing pipelines, introduce variability, and while useful and important, must be considered when training these models—something that has not been systematically investigated.

Another major challenge is the reliability, reproducibility, and generalizability of the machine learning models. Our extensive literature review demonstrates that there is a whole range of machine-learning models that have been proposed including traditional models, deep-learning models, and multimodal models. We selected three different models available as open source to test the reproducibility, and generalizability of those models [31, 45, 48]. Our ablation study (Table 3) shows that some of the models may not be reliable, and others may not be generalizable across different cohorts. Note that the three models available to us were trained on extensive ADNI data and were trained using a single modality (sMRI data). Optimization techniques, interpretability of models, and the computational demands of training complex models also add complexity to the development of accurate diagnostic tools for AD.

Our survey reviewed various neuroimaging modalities, including sMRI, fMRI, and PET, including preprocessing and data management techniques, both traditional ML and DL approaches, and a comprehensive evaluation of the models with different cohorts. This evaluation provides valuable insights into the practical applicability of these models in real-world scenarios, highlighting the challenges and potential pitfalls of using ML for AD diagnosis. As a result of this survey, a few of the recommendations and best practices are listed below. For computational scientists developing various ML models, the evaluation and experimentation with different data cohorts can broaden the scope of their studies by considering alternative datasets beyond a single (e.g., ADNI) cohort. Including datasets such as OASIS, Biomarker and Lifestyle Flagship Study of Ageing (AIBL) [129], and National Alzheimer’s Coordinating Centers (NACC) [130] could offer a more comprehensive perspective on AD diagnosis using neuroimaging and machine learning, potentially revealing variations in results and enhancing generalizability. The variability of the results (Figs. 3, 4, and 5) shows room for investigating different ML approaches beyond those covered in the survey paper. Alternative algorithms, ensemble methods, or hybrid models could be explored to enhance diagnostic accuracy and effectiveness. Validating and replicating findings across multiple datasets is crucial for enhancing the credibility and generalizability of ML models for AD diagnosis. Similarly, formulating a uniform criterion for different classes of ADRD diagnosis will help ensure that models are learning consistent information for a given label. With the current variability, the models learn different signatures for the same label, leading to inconsistencies across the board. Lastly, the evaluation of the model must be completed with no data leakage to ensure that metrics and results are consistent, reproducible, and robust.

### Future directions

While ML approaches show significant promise in advancing early AD/ADRD diagnosis, several critical challenges must be addressed to enable broader applicability in clinical settings. Below, we outline key areas for future research, with a focus on overcoming existing barriers and driving meaningful advancements in the field.

#### Improving clinical interpretability

One of the primary barriers to the adoption of ML models in clinical practice is their lack of interpretability. Clinicians require models that not only provide predictions but also offer actionable insights into the underlying mechanisms driving those predictions. Future research should focus on developing interpretable ML frameworks that employ methods such as attention mechanisms, feature attribution, and SHAP (SHapley Additive exPlanations) to highlight the most critical neuroimaging biomarkers for AD diagnosis. For instance, attention mechanisms can pinpoint specific regions of interest in brain scans, while SHAP values can quantify the contribution of individual features to model predictions. Furthermore, integrating explainability frameworks into ML workflows will ensure that the outputs align with clinical decision-making processes, fostering trust and transparency among healthcare providers. Additionally, researchers should collaborate with clinicians to create interpretable visualizations and reports tailored to the needs of medical professionals, enabling them to make more informed and confident decisions based on ML model outputs.

#### Enhancing model robustness and generalizability

ML models often struggle to generalize across diverse datasets, particularly when faced with variations in demographics, imaging modalities, or acquisition protocols as shown our results in this paper. Future studies should conduct large-scale evaluations of model performance across heterogeneous datasets to assess their robustness and identify biases. Domain adaptation techniques and transfer learning offer promising solutions for mitigating cohort-specific biases and improving model performance on underrepresented populations. For example, transfer learning can leverage knowledge from well-annotated datasets to enhance performance on smaller, less-represented datasets. Additionally, researchers should explore ensemble learning methods that combine predictions from multiple models to improve robustness and reduce the risk of overfitting. A concerted effort to create benchmarks that account for demographic and modality variability will also enable a more standardized evaluation of model performance, ensuring broader applicability in real-world clinical settings.

#### Multimodal data integration

AD is a multifactorial condition, with its diagnosis and progression influenced by a combination of genetic, neuroimaging, proteomic, clinical and social determinants of health factors. Future research should prioritize the integration of multimodal data sources to improve diagnostic accuracy and the discovery of novel biomarkers. Combining sMRI, fMRI, and PET imaging with complementary data such as genetic profiles, cerebrospinal fluid biomarkers, and cognitive assessments can enable a more holistic understanding of disease mechanisms. Furthermore, researchers can consider the use of real-time health monitoring tools, such as wearable devices and electronic health records to complement neuroimaging data. These multimodal approaches can capture the dynamic nature of AD progression, allowing for earlier and more precise diagnoses. Advances in data fusion techniques, such as tensor decomposition and graph neural networks, could further facilitate the integration and analysis of multimodal datasets, unlocking new opportunities for personalized medicine.

#### Data augmentation and synthetic data generation

The scarcity of labeled neuroimaging data, particularly for underrepresented populations, poses a significant challenge for training robust ML models. Generative models, such as Generative Adversarial Networks (GANs), hold immense potential for creating realistic synthetic datasets that address class imbalance and augment limited datasets. For instance, GANs can generate synthetic MRI scans that closely resemble real-world samples, enabling researchers to train models on a more diverse range of data. This approach not only improves model performance but also preserves patient privacy by eliminating the need to share sensitive medical data. Future research should explore advanced data augmentation techniques, such as style transfer and domain-specific augmentation, to further enhance the diversity and quality of training datasets. Additionally, validating the clinical relevance of synthetic data through expert evaluation will be critical to ensure its utility in real-world applications.

#### Real-world clinical deployment

The transition from research to clinical practice requires the development of user-friendly tools and systems that integrate seamlessly into existing healthcare workflows. Future efforts should focus on designing software platforms that enable clinicians to deploy and interpret ML models with minimal technical expertise. These platforms should include intuitive interfaces, explainability features, and real-time feedback mechanisms to support clinical decision-making. Additionally, prospective clinical trials are essential to validate the effectiveness of ML-based diagnostic tools in real-world settings. These trials should evaluate not only the diagnostic accuracy of the models but also their impact on patient outcomes, care efficiency, and clinician satisfaction. Researchers should also explore scalable deployment strategies, such as cloud-based and edge-computing solutions, to ensure accessibility in resource-constrained settings, including rural or underfunded clinics. By addressing these challenges, ML models can be effectively translated into tools that improve patient care.

#### Addressing ethical and societal considerations

Ethical considerations are paramount when developing ML models for medical applications, particularly in ensuring fairness and inclusivity. Future research must prioritize the design of models that account for underrepresented populations, including those with unique demographic, geographic, and socioeconomic characteristics. For example, datasets should be curated to include diverse patient groups to mitigate biases that disproportionately affect vulnerable populations. Additionally, researchers should establish robust frameworks for handling sensitive neuroimaging and clinical data, emphasizing privacy preservation and data security. Techniques such as federated learning, which enables model training without sharing raw data, can be employed to enhance data privacy while maintaining model performance. Efforts should also focus on ensuring equitable access to ML-driven diagnostic tools, preventing disparities in healthcare delivery across different regions and communities. Transparent reporting and community engagement will be essential for fostering trust and acceptance of these technologies.

By addressing these future directions, the field of ML-driven AD diagnosis can move closer to achieving clinically viable models that improve early diagnosis, enhance patient outcomes, and reduce healthcare disparities. As ML methods continue to evolve, their integration with multimodal neuroimaging and clinical workflows holds the potential to transform the landscape of AD/ADRD diagnosis and care.

## Data Availability

No datasets were generated or analysed during the current study.
